# Tumor Immunotherapy: Lessons from Autoimmunity

**DOI:** 10.3389/fimmu.2014.00212

**Published:** 2014-05-13

**Authors:** Christian Maueröder, Luis Enrique Munoz, Ricardo Alfredo Chaurio, Martin Herrmann, Georg Schett, Christian Berens

**Affiliations:** ^1^Institute of Clinical Immunology, Department of Internal Medicine 3, University of Erlangen-Nuremberg, Erlangen, Germany; ^2^Department of Biology, University of Erlangen-Nuremberg, Erlangen, Germany

**Keywords:** immunotherapy, tumor vaccination, cell death, tumor microenvironment, autoimmunity, danger model

The sequel to the landmark article “The Hallmarks of Cancer” adds two emerging hallmarks and two enabling characteristics to the six original hallmarks (1). One emerging hallmark is the property of cancer cells to escape the immune system. Clinically apparent tumors arise as winners in a complex, hard-fought duel between cancer cell survival and eradication by the immune system.

Immunoediting, a term used for describing interactions between tumor and immune system, only occurs when, during the process of malignant transformation, cells develop features recognized by the immune system (2). The contribution of the immune system to recognition and elimination of malignant cells has been and still is being discussed controversially: some studies support the concept of immunosurveillance (3, 4), whereas others only observed small effects of the immune system in the prevention of cancer (5, 6). Recent studies suggest that, while there is evidence for immunosurveillance, not all aspects of the interaction between malignant cells and the immune system can be explained by immunoediting alone (7): some tumors never show properties making them targets of the immune system, whereas other tumors are recognized, but not eliminated due to immune suppression induced by the tumor.

However, if tumor cells are recognized as “altered cells,” their perpetual confrontation with the immune system evokes strong selection conditions favoring tumor cells that (I) lose properties making them targets of the immune system and (II) gain properties making them appear non-dangerous (8). If the tumor succeeds in decreasing its immunogenicity, it will reach a stage when the immune system does not consider those cells to be “altered-self” anymore. The tumor is now perceived as “self” and non-dangerous, with all privileges of normal healthy tissues.

When we think about therapies that elicit anti-tumor responses at this stage, we actually have to think about re-creating and enforcing tumor recognition, because, malignant tissues, although having been infiltrated by T-effector lymphocytes and, thus, being recognized by the immune system, frequently do not show remission. This correlates with reports that recruitment of T-effector lymphocytes to the site of the tumor is not necessarily sufficient for its eradication and that tumor immunity heavily depends on breaking tumor tolerance, i.e., by depletion of T-regulatory lymphocytes or by shielding T-effector lymphocytes from immune-suppressive molecules like PD-L1 (9). We propose that the need for inducing immunity and breaking of tolerance might be akin to activating some kind of tumor-specific (auto)-immunity.

The ideal tumor therapy results in local control of the primary tumor, systemic control of potential metastases and triggers an anti-tumor immune response ultimately leading to the elimination of all malignant cells. To achieve this, tumor therapy needs to deal with the problem that the immune system does not consider the tumor being dangerous anymore – it has been adopted as “self-organ.” Consequently, tumor therapy should focus on making the immune system aware of this hidden danger.

This concept was first put into practice by William Coley, who injected a cocktail of dead bacteria into tumors in the late 1800s, achieving cures in ≈30% of his patients with sarcoma and lymphoma (10, 11). The mechanism responsible for this seems to be LPS-induced IL-12 secretion triggering a robust bystander T_h_1-response against the tumor cells (12). Likewise, an attenuated *Salmonella* vaccine can induce a shift in the tumor milieu from an immune-suppressive to an immunogenic microenvironment (13). The most successful application derived from Coley’s work is treatment of bladder cancer with the Bacillus Calmette–Guerin vaccine: it has become the standard therapy for superficial bladder cancer, eradicating existing tumors, reducing the frequency of tumor recurrence, delaying stage progression, and increasing survival (14). The advantage of such strategies is their lack of specificity. The immune response is not restricted to a single and, most likely, highly specific and selectable “tumor-antigen,” but the presence of danger signals at the site of the tumor “uncloaks” the cancer cells, turning them into broad range immune targets. At this point, we can exploit a mechanism, which causes a break in self-tolerance in autoimmune diseases: transient autoimmunity accompanying any inflammatory process can, in the context of steady exposure to auto-antigens and danger signals, develop into stable autoimmunity. Following Polly Matzinger’s ideas, the key to success of danger-based tumor vaccination strategies rests on repeated administration of the vaccine (15). Repeated immunization should help overcome transient tumor immunity and establish persistent protection.

One danger-based tumor vaccination approach conducts the immunization with dying tumor cells (16, 17). Certain kinds of dying or dead cells can trigger immune responses under the right conditions. The potential of dying/dead cells to induce autoimmunity can be seen in “systemic lupus erythematosus” (SLE), a chronic inflammatory disease, in which defective clearance of apoptotic cells leads to the accumulation of secondary necrotic cells, the release of danger signals, the presentation of auto-antigens and, finally, a chronic break in self-tolerance (18–20). Based on these observations, one can assume that, under the appropriate conditions, entities once considered to be non-dangerous can become re-considered dangerous. We propose that one can learn from the processes which cause breaks of self-tolerance in patients with SLE and try to harness them to induce tumor (auto-) immunity.

In the context of tumor immunology, cell death is a double-edged sword. Tumor cells often modulate apoptotic pathways rendering them less responsive to death stimuli. Down-regulation of Fas expression or resistance to Fas-mediated apoptosis are common strategies of tumor cells to escape immunosurveillance (21) and are associated with resistance to therapy, metastatic capacity, and poor prognosis. For example, c-Jun and Stat-3 act as oncogenes by cooperatively repressing the transcription of *Fas*, rendering tumor cells insensitive to FasL-induced apoptosis (22). A complete loss of Fas expression is less common, possibly to low-level expression of Fas supporting tumor growth (23). Many other mechanisms to evade elimination by apoptosis, i.e., suppression of caspase-8 activity by CDK1/CYCLIN B1 dependent phosphorylation (24), *bcl-2* amplification (25), and loss of pro-apoptotic proteins like BAX (26) and PUMA (27), have been reported for a large variety of cancer types (28).

These findings are hard to reconcile with the observation that a high rate of tumor cell apoptosis is accompanied by poor prognosis in some types of cancer (29–31). It is known that cancer cells show many different changes to the apoptotic machinery (28, 32); but does this mean they have lost all capability to execute apoptosis? Apoptosis is necessary for tissue homeostasis, contributes to the maintenance of peripheral tolerance and might even play a role in the induction of the latter (33, 34). The fact that most chemotherapeutics at least initially induce tumor apoptosis confirms that cancer cells frequently retain their ability to execute apoptosis (35, 36). It is reasonable to assume that those parts of the apoptotic machinery involved in the induction of extrinsic apoptosis by the immune system preferentially experience negative selection. If other parts of the apoptotic pathway would also be a potential source of harm, why do they, in defiance of the exceptional adaptability of cancer cells, still function properly? We suggest that, in contrast to the oversimplified illustration, cancer cells do not completely lose their capability to undergo apoptosis, but that their apoptotic machinery can instead be “hijacked” in a way that not only sustains their existence, but also accelerates tumor formation (37–39): an “altruistic” death of limited amounts of cancer cells is a possible way to support the survival of the tumor on the whole.

Over the years, the tumor-supportive effects of apoptotic tumor cells have received greater recognition, and it is now assumed that apoptotic tumor cells and the corresponding phagocytes participate in forming and shaping the tumor microenvironment (40). Apoptotic cells release a diverse spectrum of molecules, which act as “keep-out,” “find-me,” “eat-me,” and “tolerate-me” signals and ensure that the clearance of apoptotic cells is facilitated by defined groups of phagocytes, in particular by macrophages (41).

Of particular interest are lipid mediators, which are released from cells undergoing apoptosis: (I) lysophosphatidylcholine is a potent chemoattractant for macrophages and is released from cells executing apoptosis (42). (II) Upon proteolytic activation of sphingosine kinase 2, sphingosine-1-phosphate (S1P) is released from apoptotic cells (43). In addition to its role as a chemoattractant (44), S1P polarizes macrophages toward a non-inflammatory phenotype (M2), characterized by decreased secretion of TNF-α and IL-12-p70 and increased formation of IL-8 and Il-10 (45).

The engulfment of apoptotic cells by macrophages induces their polarization toward the M2-phenotype (Figure 1A). These alternatively activated macrophages tune down inflammation and promote angiogenesis, tissue remodeling, and repair (46, 47). Furthermore, phagocytosis of apoptotic cells by M1-macrophages also triggers a shift toward alternative activation (48). Fittingly, a large number of macrophages at the site of the tumor are associated with a poor prognosis and these tumor-associated macrophages share many characteristics with M2-macrophages (49, 50). Their presence at the site of a tumor supports Dvorak’s concept that tumors are “wounds that do not heal” (51).

**Figure 1:**
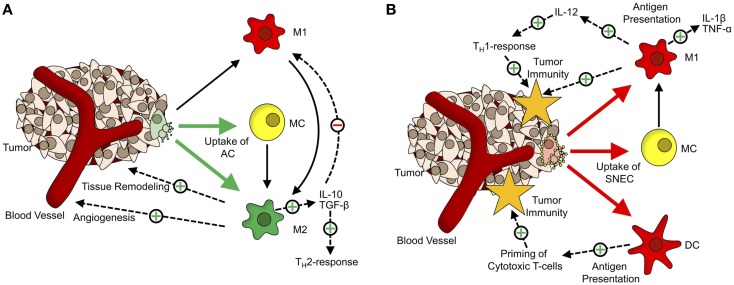
**The dual role of cell death in tumor tolerance/immunity**. **(A)** Role of apoptotic cells in formation of the tumor microenvironment. Apoptotic cells (AC) are mainly taken up by monocytes (MC; yellow) and alternatively activated macrophages (M2; green). Upon phagocytosis of ACs, MCs, and classically activated macrophages (M1, red) get polarized toward an M2-phenotype. M2-macrophages participate in tissue remodeling and angiogenesis and via secretion of anti-inflammatory cytokines (TGF-β, IL-10), inhibit M1-activation of macrophages and shift T_H_1-responses toward the T_H_2-phenotype. **(B)** Tumor-supportive effects of apoptotic cells are abrogated by Annexin-A5. Annexin-A5 (yellow circles on secondary necrotic cells) inhibits swift clearance of apoptotic cells, leading to progression of ACs into secondary necrosis. Secondary necrotic cells (SNEC) are mainly taken up by MCs, classically activated macrophages and dendritic cells (DC; red). Upon phagocytosis of SNEC, MCs get polarized toward the M1-phenotype. Phagocytosis of SNEC by DCs leads to antigen presentation and priming of T cells. Classically activated macrophages secrete inflammatory cytokines (TNF-α, IL-1β) and induce T_H_1-responses via IL-12.

In line with these findings is the observation that inhibiting the clearance of apoptotic tumor cells by administration of Annexin-A5 retards tumor growth in a colorectal carcinoma model and greatly enhances the effect of immunization with irradiated lymphoma cells in a lymphoma model (52, 53). The data presented suggests that this is due to the fact that the non-inflammatory clearance of apoptotic cells by macrophages is blocked so that the apoptotic cells get secondarily necrotic. The concomitant loss of membrane integrity is accompanied by the release of danger-associated molecular patterns (DAMP), which act as natural adjuvants. Phagocytosis of secondary necrotic cells by macrophages (Figure 1B) leads to an increased expression of TNF-α and IL-1β. In addition, several DAMPs released from secondary necrotic cells, like HMGB1 and HMGN1, are potent stimuli for dendritic cell maturation (54).

The close interaction between tumors, the immune system and cell death gives rise to new therapeutic approaches. Some aspects of this interaction may be exploited to support conventional cancer therapies. Systemic administration of Annexin-A5 or other phosphatidylserine ligands may help slow down tumor progression by blocking the tumor-supportive properties of apoptotic cells. In combination with radio- or chemotherapy, Annexin-A5 could be used as a natural adjuvant, which increases the immunogenicity of dying tumor cells and, thus, helps elicit an anti-tumor immune response (55). This may be especially helpful in targeting cancer cells, which have resisted therapy and would possibly lead to a relapse.

Until recently, cell death was either characterized as programed and apoptotic, or accidental and necrotic. This paradigm has been undermined by the discovery of several other forms of cell death, ranging from immunogenic apoptosis (56) or necroptosis (57) to pyroptosis (58, 59). So, in addition to manipulating cell death induced by radio- or chemotherapy in a way to increase its immunogenicity, the direct induction of immunogenic tumor cell death pathways might become a promising approach in cancer therapy (17, 54, 60), especially, since our means of controlling the manner of cell death have greatly increased during recent years (61–63).

Surgical removal of malignant tissue plays an important role in modern cancer therapy. The cancer cells obtained in this process may be used as a vaccine to establish anti-tumor immunity, if treated and administered properly. The focus must be on cancer cells dying by immunostimulatory forms of cell death leading to necrotic cell corpses, whose deployment would activate antigen-presenting-cells. This way, the specific autologous tumor cells can serve as reservoirs of tumor antigens, which, upon phagocytosis by inflammatory macrophages and dendritic cells, are effectively (cross-)presented. The impact of the vaccine could be optimized by repeated administration of the dying cells. However, we have to be very careful, since a recent study indicates that excessive immune responses against cancer can result in an increased risk of developing the autoimmune disease scleroderma (64), pointing out several parallels between the induction of autoimmunity and immunosurveillance. While this study actually supports the idea that mechanisms inducing autoimmunity can also be used to elicit tumor immunity, it also suggests that any agents used to recruit anti-tumor responses must be well-balanced. After all, nobody wants to escape cancer’s fire by jumping into the frying pan of autoimmunity.

## Conflict of Interest Statement

The authors declare that the research was conducted in the absence of any commercial or financial relationships that could be construed as a potential conflict of interest.
